# Influence of Polyvinyl Alcohol (PVA) on PVA-Poly-*N*-hydroxyethyl-aspartamide (PVA-PHEA) Microcrystalline Solid Dispersion Films

**DOI:** 10.1208/s12249-020-01811-z

**Published:** 2020-10-02

**Authors:** Zahra Al-Sahaf, Bahijja Raimi-Abraham, Mariano Licciardi, Laura Modica de Mohac

**Affiliations:** 1 King’s College London, London, UK; 2 University of Study of Palermo, Palermo, Italy

**Keywords:** buccal film, PVA, PHEA, ibuprofen sodium, crystalline

## Abstract

This study was conducted to formulate buccal films consisting of polyvinyl alcohol (PVA) and poly-*N*-hydroxyethyl-aspartamide (PHEA), to improve the dissolution of the drug through the oral mucosa. Ibuprofen sodium salt was used as a model drug, and the buccal film was expected to enhance its dissolution rate. Two different concentrations of PVA (5% w/v and 7.5% w/v) were used. Solvent casting was used to prepare films, where a solution consisting of drug and polymer was cast and allowed to dry. Attenuated total reflection Fourier transform infrared spectroscopy (ATR-FTIR), differential scanning calorimetry (DSC), and scanning electron microscopy (SEM) were used to investigate the properties of films. *In vitro* dissolution studies were also conducted to investigate drug release. SEM studies showed that films containing a higher concentration of PVA had larger particles in microrange. FTIR studies confirmed the presence of the drug in films and indicated that ibuprofen sodium did not react with polymers. DSC studies confirmed the crystalline form of ibuprofen sodium when incorporated within films. *In vitro* dissolution studies found that the dissolution percentage of ibuprofen sodium alone was increased when incorporated within the film from 59 to 74%. This study led to the development of solid microcrystalline dispersion as a buccal film with a faster dissolution rate than the drug alone overcoming problem of poor solubility.

## INTRODUCTION

Tablets represent the most common dosage form available in the market, but they present both pharmaceutical and clinical issues (1). Tablet compression is compromised when using a hygroscopic drug with low density (2,3). Moreover, drugs with low solubility, which are 60% of marketed compounds, would have a low dissolution into the gastrointestinal tract, causing a reduced bioavailability (4). Even if an oral tablet denotes easy administration for most of the patients, it represents a cause of discomfort for patients with swallowing aversion, pediatric and geriatric population, or with altered absorption diseases that do not benefit from conventional oral route (5–7). Some of the factors associated with low compliance are side effects experienced by the patient and the complexity of treatment, such as multiple dosing times every day (8). Therefore, tablets are not always preferred route of administration.

Oral cavity film administration has recently emerged as a promising alternative to solid dosage forms (9). Drug delivery *via* the oral cavity can take place by placing film under the tongue or placing the film on inner cheek allowing rapid absorption of the drug and is, therefore, suitable for fast-release formulations (10). An important factor influencing oral cavity drug delivery is lack of keratinization in cheek mucosa and sublingual regions resulting in high permeability and systemic delivery (11,12). Therefore, patches applied to buccal mucosa have a potential advantage to by-pass first-pass metabolism leading to an increase in the bioavailability (7,13,14). Furthermore, in comparison to oral tablets, films are ultrathin, flexible, and tend to be less obtrusive and therefore more acceptable (15).

Solvent casting is a prominent method used to prepare solid dispersion as an oral film where the cast solution is dried and cut into the desired size (16). This method is widely used to improve the dissolution of poorly water-soluble drugs and, therefore, to enhance their bioavailability in the body (17). Pharmaceutical film improvement in dissolution rate is often due to the use of hydrophilic polymers which cause an increase in solubilization effect of carrier (18). Furthermore, solid dispersions lead to a drug particle size reduction, improving drug wettability (19). In solid dispersion formulation, a key element is the morphological structure of compounds that could be either amorphous or crystalline affecting release profile of API from formulation (20). Modica de Mohac *et al.*, in 2020, described as the different morphological structure could influence release profile and, therefore, important in polymer selection while improvement in release profile is sought (21). Mainly, crystalline and amorphous drugs present different enthalpy, entropy, and free energy that affect stability and dissolution rate of final dosage form (22). Amorphous materials show weaker attractive intermolecular forces that are more easily broken compared to crystalline counterparts, resulting in more soluble material and having a faster dissolution rate (20). However, several studies have shown that the formulation of solid microcrystalline dispersions could both improve dissolution rate and dosage form stability (16,23).

The overall aim of this study was to formulate a solid microcrystalline dispersion as a pharmaceutical film to investigate the effect of the combination of polyvinyl alcohol (PVA) and poly-*N*-hydroxyethyl-aspartamide (PHEA) concentration to increase drug dissolution profile by maintaining its crystalline state. PVA was selected as based polymer due to its water solubility and excellent film-forming properties (24). Furthermore, it is odorless and non-toxic. PVA was combined with novel polymer PHEA (25–27) that has many attractive properties, such as water solubility and absence of toxicity (28). Additionally, it is biodegradable and was used previously in drug delivery systems (29). PHEA is also very hydrophilic and has a high degree of mucoadhesion when formulated at 2.5% w/v in combination with PVA at 5% w/v (30). Present work aimed to prove that increasing concentration of PVA in conjunction with new polymer PHEA would allow obtaining a fast-release profile. Ibuprofen sodium was used as model drugs due to its low solubility of 0.0219 mg/mL (31). The oral film was produced *via* solvent casting and film physicochemical properties were characterized using attenuated total reflection Fourier transform infrared spectroscopy (ATR­FTIR), differential scanning calorimetry (DSC), and scanning electron microscopy (SEM). *In vitro* dissolution studies mimicking oral cavity were also conducted.

## MATERIALS AND METHODS

### Materials

Ibuprofen sodium salt [> 98%] purchased from Sigma-Aldrich, PVA [molecular weight (Mw) = 146,000–186,000 g/mol, 87–89% hydrolyzed] purchased from Sigma-Aldrich, and PHEA was synthesized in lab previously (32,33). Buffer tablets pH 6.8 and ethanol were purchased from Sigma-Aldrich.

### Sample Preparation: Aqueous Solution

Aqueous solutions of PVA with a concentration of 5% w/v and 7.5% w/v were prepared by stirring PVA in distilled water using a magnetic stirrer. Distilled water was heated at a temperature of 90°C, and PVA granules were added and stirred for 2 h at 80°C. PHEA at a concentration of 2.5% w/v was added to already made PVA solutions and stirred for 12 h using a magnetic stirrer. Ibuprofen sodium at a level of 10% w/v was then added to PVA and PHEA solution and stirred for 2 h.

### Sample Preparation of Pharmaceutical Films

The pharmaceutical films were prepared by casting 5 mL of the solution, described in “Sample Preparation: Aqueous Solution,” in a 60-mm plastic petri dish and placing petri dishes in a fume cupboard for 48 h to allow the solvent to evaporate. After 48 h, the film was dried and was subsequently peeled off gently from petri dish using forceps. Films were then cut into 1 cm × 1 cm square pieces. Films were produced according to the ratio reported in Table I.

**Table I:** Concentration of PVA, PHEA, and Ibuprofen Sodium Used to Prepare Pharmaceutical Films

Film	PVA (% w/v)	PHEA (% w/v)	Ibuprofen sodium (% w/v)
F1	5.0	–	–
F2	7.5	–	–
F3	5.0	2.5	–
F4	7.5	2.5	–
F5	5.0	2.5	10.0
F6	7.5	2.5	10.0

*PVA* polyvinyl alcohol, *PHEA* poly-N-hydroxyethyl-aspartamide

### Scanning Electron Microscopy

SEM studies were conducted on F5 and F6 films to analyze the morphology of particles and to calculate particle size. Images were collected on 1 cm × 1 cm film section using a Hitachi S5000 Field Emission Gun SEM with a tungsten tip. Before analysis, film samples were coated with 10 nm of gold. All images were taken using the secondary electron detector. ImageJ software was used to calculate the average particle size of drug particles by taking 100 measurements. Particle distribution index (PDI) was calculated accordingly with the following formula: (PDI) = <*d*^2^> / <*d* >^2^ (34).

### Attenuated Total Reflection Fourier Transform Infrared Spectroscopy

FTIR analysis was conducted using a Perkin Elmer Frontier FTIR spectrometer to identify any interactions occurring between molecules, such as hydrogen bonding. Diamond ATR crystal was wiped clean with ethanol, and a background scan was carried out. The sample was then placed onto the crystal. The pressure was applied at a force gauge of 70. A total of 16 scans were taken at a resolution of 2 cm^−1^ and using a wavelength range of 4000–600 cm^−1^. Two replicates were carried out for each sample.

### Differential Scanning Calorimetry

DSC analysis was conducted using a DSC 2920 machine (TA instruments) to obtain melting temperature and determine whether materials are present in an amorphous or crystalline form. DSC analysis was conducted at a rate of 5°C/min, and samples were heated to 250°C. Nitrogen purge gas used had a flow rate of 130 mL/min. Samples were also held isothermally for 5 min. The sample was weighed and placed inside aluminum pans, and the lid was crimped to ensure it was securely closed. Pan was set in DSC instrument with an empty pan as a reference. Two replicates were carried out for each sample.

### Drug Content Studies

UV analysis was used to produce a calibration curve of known concentrations of ibuprofen sodium. Absorbance values were measured using a Perkin Elmer Lambda 35 UV/VIS spectrometer. 0.05 g of ibuprofen sodium was weighed and dissolved in 100 mL of ethanol to form a stock solution, then diluted. A small volume of each solution was placed in a 4-mL quartz cuvette to measure absorbance values at a wavelength of 263 nm and obtain a calibration curve. Drug content was determined in 1 cm × 1 cm film samples that were weighed and dissolved in 10 mL of ethanol. Film samples with a weight of approximately 0.03 g were used. Samples were left in ethanol for 3 days, and the solution was then filtered through a 0.45-μm filter. Samples were then analyzed spectrophotometrically at 263 nm to determine drug content.

### *In Vitro* Dissolution Studies

Drug release from the film was investigated through dissolution studies; those were conducted using 100 mL of buffer pH 6.8 solution as a dissolution media. A paddle apparatus was used for this study, and dissolution media was heated to 37°C using a magnetic stirrer and stirred at 100 rpm. A 1 cm × 1 cm film section was weighed and placed in dissolution media. Film samples with a weight of approximately 0.03 g were used. At set time intervals, 10 mL was drawn out and replaced with 10 mL of same dissolution media at the same temperature. Time intervals used were 0, 2, 5, 10, 15, 20, 25, 30, 35, 40, 45, 60, 120, and 180 min. Solutions were then analyzed spectrophotometrically at 263 nm. Three replicates were carried out for dissolution of F5 and F6 films, and an average was calculated. Same dissolution method was used to carry out dissolution studies for ibuprofen sodium alone. Weight of ibuprofen sodium used was approximately 0.002 g. Statistical analysis was performed using two-way ANOVA to calculate the *p* value.

## RESULTS

### Physicochemical Characterization

**Fig. 1:**
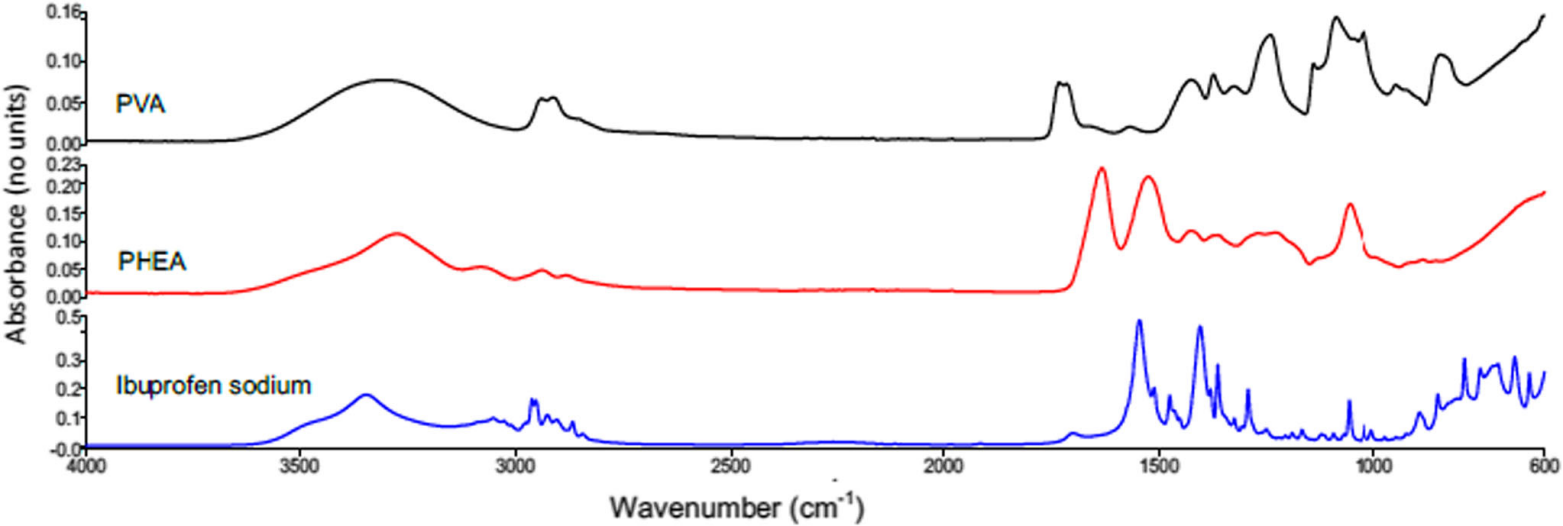
ATR-FTIR spectra of raw materials PVA, PHEA, and ibuprofen sodium

At first, physical-chemical properties of raw material were characterized to investigate changing in drug solid state after formulation. ATR-FTIR was used to assess main peaks related to PVA, PHEA, and ibuprofen sodium, respectively. Notably, this method allows identification of creation of hydrogen bonding that might indicate the formation of new bonds between drug and polymers (35). Figure 1 presents ATR-FTIR spectra for raw material and peak assignation was reported in Table II. Peak assignation was coherent with literature, and ibuprofen sodium spectra resulted in being consistent with pure drug spectra in the crystalline form (36–38).

**Table II:** Peak Assignation for Each Raw Material PVA, PHEA, and Ibuprofen Sodium. Data Are Coherent with Literature Findings (36–38)

Raw material	Peaks	Assignation
PVA	3290 cm^−1^	Stretching O–H group
	2939 cm^−1^	Asymmetric stretching C–H group
	1732 cm^−1^	Stretching C=O
	1567 cm^−1^	Stretching C=C
	1421 cm^−1^	Symmetric bending CH_2_ group
PHEA	3276 cm^−1^	Stretching O–H bond
	2938 cm^−1^	Aliphatic stretching C–H group
	1633 cm^−1^	Stretching C=O group
	1525 cm^−1^	Vibrations N–H group
	1427 cm^−1^	Aliphatic bending C–H group
	1362 cm^−1^	Bending O–H group
	1056 cm^−1^	Ester group
Ibuprofen sodium	2951 cm^−1^	Asymmetric stretching CH_3_ group
	1698 cm^−1^	Stretching C=O group
	1545 cm^−1^	Stretching vibrations C=C
	1251 cm^−1^	C–O stretching
	748 cm^−1^	Vibration CH_2_ group

*PVA* polyvinyl alcohol, *PHEA* poly-N-hydroxyethyl-aspartamide

Drug solid-state morphology is an essential parameter to evaluate to understand the dissolution profile. DSC analyzed thermal properties of drug and polymers, and thermograph of PVA showed an endothermic peak at 192°C recognized to be the melting point of polymer which is present in crystalline form. This finding relates to the literature (39), while PHEA was amorphous and no peak is visible at DSC. Ibuprofen sodium showed a sharp peak at 101°C (Δcp 10.89 J/g), confirmed by existing literature, which indicates that it is present in its crystalline form (31).

**Fig. 2:**
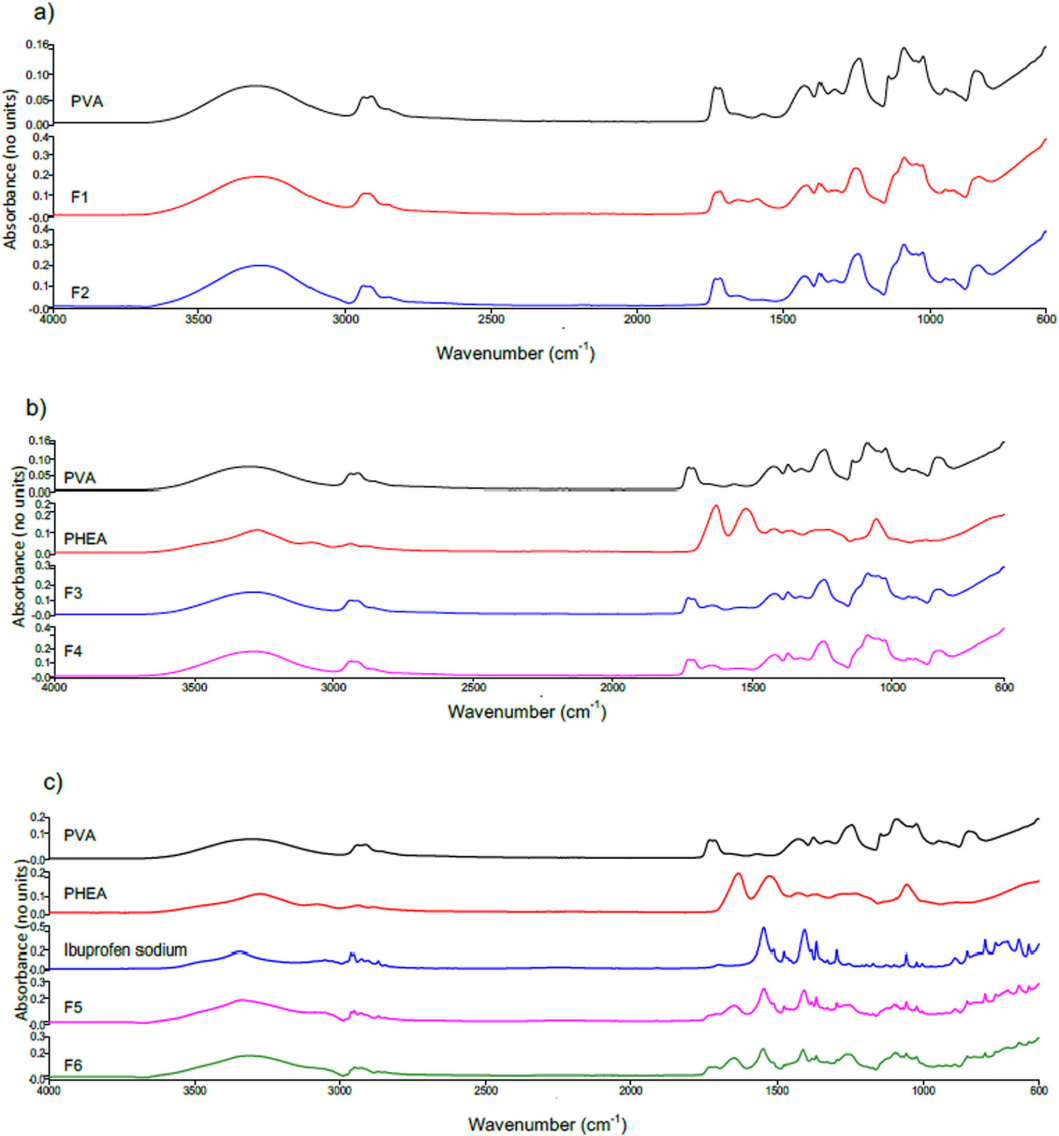
FTIR spectra of **a** PVA with F1 and F2; **b** PVA and PHEA with F3 and F4; and **c** PVA, PHEA, and ibuprofen sodium with F5 and F6

Once patches were formulated, they were evaluated for the presence of a hydrogen bond between polymers and drug to assess the formation of interactions that might occur during the formulation process. Spectra are shown in Fig. 2. ATR-FTIR spectra of pure PVA and films containing only PVA and water (F1 and F2) were consistent with pure PVA spectra, and no water was detected. In formulations F3 and F4, there were observed peaks at 3284 cm^−1^ for F3 and 2939 cm^−1^ for F4, respectively assigned to O–H group and C–H group in PVA. It was also noticed that the presence of peaks at 1633 cm^−1^ and 1056 cm^−1^ both related to PHEA chemical structure. But no new peak was observed demonstrating no hydrogen bond formation. In ATR-FTIR spectra of F5 and F6, there were characteristic peaks of ibuprofen sodium which were seen. For instance, a peak was observed at 2950 cm^−1^ in F5 film due to the asymmetric stretching of the CH3 group. Also, a peak was observed at 1647 cm^−1^, which was assigned to stretching of C=O group. Fingerprint region of F5 and F6 films showed similar activity to raw ibuprofen sodium with peaks at 1252 cm^−1^ for F6 film which is apportioned to C–O stretching. Also, a peak was highlighted at 749 cm^−1^ of F5 film which is assigned to vibration of CH_2_ group. No peaks referring to the formation of new bonding were identified.

**Fig. 3:**
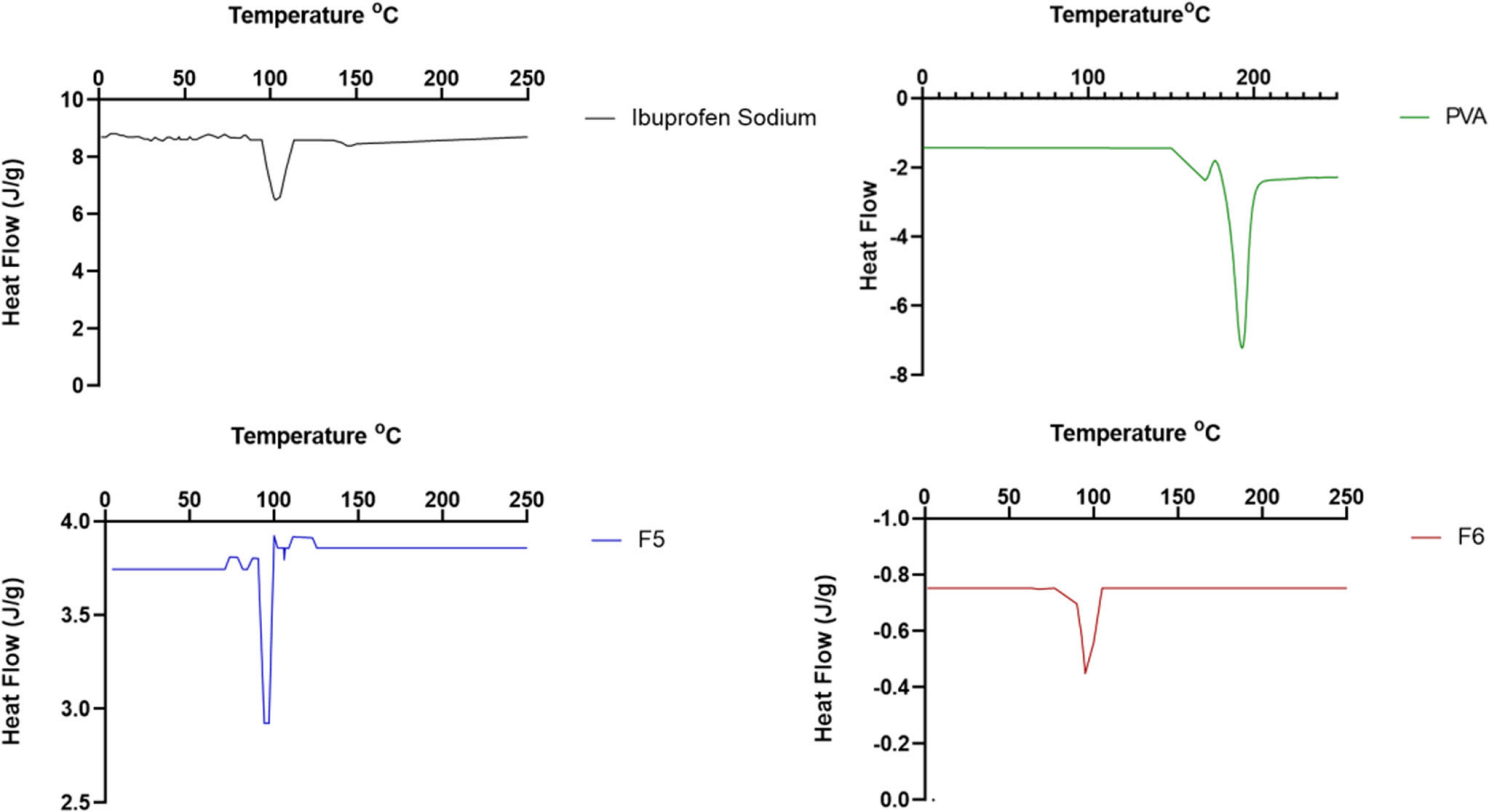
DSC graphs of ibuprofen sodium and pure PVA compared with F5 and F6 that present respectively peaks at 96°C and 95°C (endotherm down)

Therefore, drug morphology within formulations was investigated as it is a well-known effect on solid state on dissolution profile (40). DSC thermographs for F1, F2, F3, and F4 film showed endothermic peaks at 191°C, 189°C, 191°C, and 189°C, respectively. These peaks correspond to the melting temperature of PVA, confirming the crystalline form of PVA in films. Figure 3 shows the thermographs of formulations F5 and F6. Mainly, F5 film showed an endothermic peak at 96°C (Δcp 0.73 J/g), and F6 film showed an endothermic peak at 95°C (0.68 J/g). These two peaks correspond to the melting point of ibuprofen sodium and highlight the crystalline form. PVA crystalline peak, which is reported in Fig. 3, was not identified in F5 and F6, suggesting PVA amorphization. Confirmation about drug crystallinity could be sought to be identified, in the previous paragraph, of the main peak of ibuprofen sodium as a crystalline molecule. Moreover, it was observed that heat capacity (Δcp) of each formulation, F5 and F6, is respectively 7.5% and 6.98% of Δcp of ibuprofen alone, and this value is coherent with drug loading found for both formulations and described below.

**Fig. 4:**
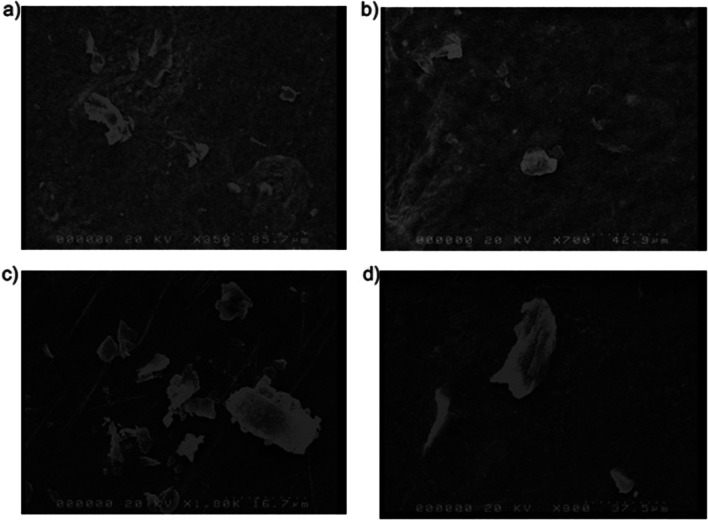
SEM images of F5 with a magnification of **a** × 350 and **b** × 700 and F6 with a magnification of **c** × 1K and **d** × 900

Later, the effect of different concentrations of PVA on particle size was investigated. SEM images indicate that formulation containing 7.5% (w/v) of PVA presents particle diameter of 30.05 ± 3.43 μm, a 1.38-fold increase (*p* < 0.0001) with respect to particles obtained with 5.0% % (w/v) of PVA with an average diameter of 21.68 ± 4.09 μm. Even if PVA usually reduces drug particle size when used as a single polymer, it is well known that it causes drug particle growth when combined with other polymers (41,42). In this study, PVA and PHEA combination lead to obtaining an overall particle diameter that was smaller than 200 μm, reported to be typical of PVA formulation (43). Particularly, increased particle size could be due to salting out of sodium increasing interaction between ibuprofen molecules. This event is recognized as happening when two soluble polymers are dissolved at higher concentration (44). Figure 4 shows SEM images of F5 and F6 of samples with two different magnifications. For each formulation, PDI was calculated and found to be respectively 1.08 and 1.12. This result shows that the drug was not monodispersed due to formation of aggregate that is noticeable in Fig. 4 (45).

### Drug Loading Evaluation and Dissolution Study

Ibuprofen sodium drug loading was evaluated for each formulation. Results showed that ibuprofen sodium loading in film F5 was 6.82% w/v ± 0.01 and 7.01% w/v ± 0.01 in film F6. Drug loading was expected to be 10% w/v, and F5 and F6 saw ~ 69% drug loading efficacy. The lower than anticipated drug content is expected when no stabilizing agents are added to a formulation (46). The *in vitro* dissolution profile of ibuprofen sodium was analyzed in PBS pH 6.8 to mimic buccal conditions and compared with the release profile of the drug from the film formulation. The dissolution rate percentage was calculated according to assessed drug loading and related to film weight. Data showed a slower dissolution rate with ibuprofen sodium alone compared to dissolution rate when incorporated within the film. In each profile, it was observed that drug in its crystalline state achieved a condition of supersaturation due to the solubilizing effect of polymer combination (47,48). The dissolution profile of ibuprofen sodium showed a drug release of 59% at 15 min, while F5 had a drug release of 74%, a 1.25-fold increase (*p* = 0.002), and F6 of 72%, a 1.22-fold increase (*p* < 0.0001), resulting in an immediate-like release. This data indicates that the dissolution rate was faster when the drug was incorporated into films compared to medication alone. The calculation of data also confirmed area under curve (AUC) which was increment from 7093 ± 179 μg/h/mL for ibuprofen sodium to 7922 ± 443.8 μg/h/mL (F5) and 8296 ± 185.1 μg/h/mL (F6). Moreover, data showed that lower concentration of PVA increases release profile as confirmed from previous literature findings (44). As the difference between two PVA concentrations is 1.02-fold difference, it is considered the possibility that an increase in drug dissolution profile is due to the addition of PHEA itself.

## DISCUSSION

The main aim of this study was to formulate a pharmaceutical film consisting of PVA and PHEA, using ibuprofen sodium as a model drug where an improvement of drug dissolution profile was expected. Furthermore, the effect of varying concentrations of PVA on film properties was examined. At first, physical-chemical characteristics of film were analyzed. Through SEM results, some differences in particle sizes of F5 and F6 films were highlighted. F5 film, which contained a lower concentration of PVA, had smaller particles compared to F6 film, which had a higher concentration of PVA and larger particles. Budhian *et al.* also found that increasing polymer concentration caused an increase in particle size (49), which further supports the findings of this study. According to literature, PVA should show a smooth, dense surface (50), while ibuprofen sodium should appear as smooth-surfaced rectangular crystalline structures (51). In SEM images obtained, it can be observed that particles had quite an irregular shape due to aggregation, which could also lead to differences in particle size.

When formulating a drug to propose a novel drug delivery system, it is essential to investigate the presence of a bond between drug and polymer. ATR-FTIR spectra F5 and F6 with pure ibuprofen sodium showed main peaks of drug and no significant shifts of peaks which confirms that drug was incorporated within films. Drug morphology and particle size used to affect drug dissolution profile once formulated (22,52). DSC results obtained (Fig. 3) highlighted that PVA and ibuprofen sodium were maintained in crystalline forms. DSC thermographs of ibuprofen sodium showed a sharp endothermic peak at 101°C, and this was assigned to the melting of ibuprofen sodium crystals (53). These findings suggest that ibuprofen sodium solid state was unchanged during the formulation process by the presence of polymers showing lack of strong interactions between drug and polymers within the solid dispersion (54). Baek *et al.* conducted a study using an ibuprofen-loaded solid dispersion and also found that ibuprofen remained in an unaltered crystalline state after being incorporated within polymers (54). Drug remaining in a crystalline state is vital when formulating a solid microcrystalline dispersion due to the crystalline state being a thermodynamically favored state which makes it more stable than the amorphous state (55). The amorphous state has higher internal energy which can lead to higher dissolution rates but lower stability in terms of shelf-life and dissolution study reproducibility (56). However, not all studies have found results which support enhanced dissolution rate of the amorphous state. For instance, Jensen *et al.* found that crystalline carbamazepine had a faster dissolution rate than the amorphous form of carbamazepine (56). These conflicting results may be due to the unstable nature of amorphous compounds which can lead to recrystallization back into the crystalline state during storage or processing (57). This results in a loss of dissolution and solubility advantage of the amorphous state. Mainly, an amorphous dosage form of ibuprofen sodium was proved to have long-term stability issue and alteration of dissolution profile as determined by Mantas *et al.* (58).

The results from drug content studies indicate that the F5 film contained 6.82% of the drug, while F6 film contained 7.01% of the drug. This result was lower than the content of medicine expected to be in the film (10%). Small drug content could be due to method of preparation of drug content studies, which could have influenced content uniformity of films in experimental conditions used and is registered when the formulation is not provided with a stabilizer (19). Moreover, evaluation of PDI demonstrated that particles were not monodispersed in the polymer matrix. Woertz *et al.* conducted content uniformity studies on loperamide films. They found that some of the films had lower drug content than expected which could be attributed to numerous reasons, such as the concentration of polymer and drug used in films (59).

**Fig. 5:**
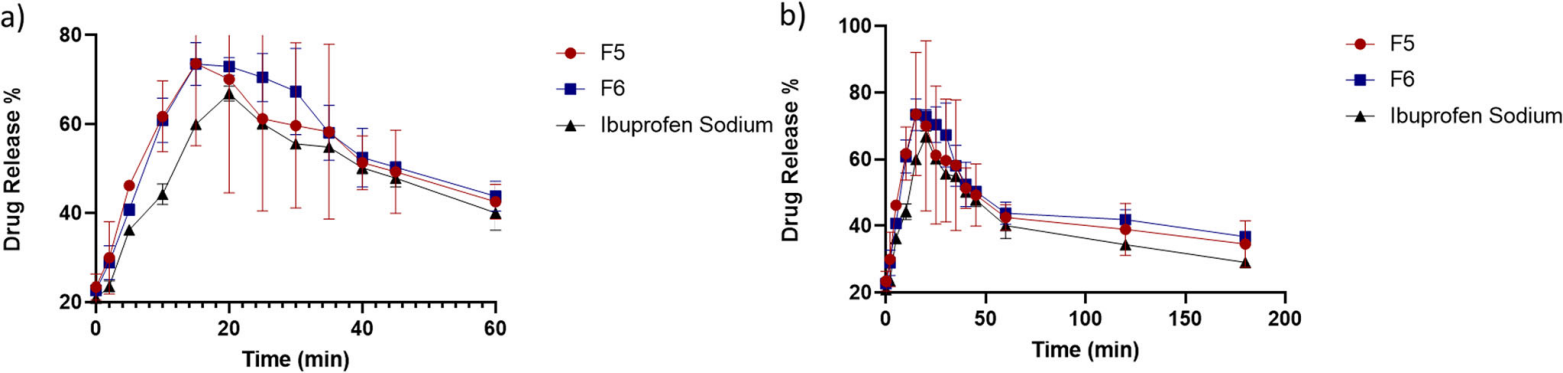
Dissolution profile of films and ibuprofen sodium **a** zoomed-in section of the first 60 min of dissolution and **b** full dissolution

The literature stated that the dissolution rate of poorly soluble drugs improves when the drug is incorporated within polymeric carriers (60). This is due to a solid dispersion forming where the presence of hydrophilic polymers has several effects such as decreasing particle size of drug (61), which means the surface area is more extensive. The diffusion layer of each particle decreases in thickness which leads to a higher dissolution rate (52). Additionally, the dispersion of a drug within polymers is expected to increase drug wettability and also prevent drug aggregation, which will also lead to a faster dissolution rate (61). Increased drug wettability caused by water-soluble polymers is because of each drug crystal that is surrounded by soluble polymeric carriers which can readily dissolve and wet drug particle surface, which increases dissolution rate (62). Results obtained from *in vitro* dissolution studies (Fig. 5) confirmed this hypothesis as dissolution rate of ibuprofen sodium alone was slower, in comparison to dissolution rate when incorporated within the film. This was highlighted in drug release profile that at 15 min was 59%. When comparing this to F5 and F6 films, maximum drug release was 74% and 72% respectively. Dissolution profiles obtained also show a “spring and parachute,” where the pattern has a “spring” which is the initial dissolution of drug and then a “parachute” which refers to prolonged supersaturation of drug (63). Besides, F5 film had a faster dissolution rate due to smaller particles, which will increase surface area for dissolution (61).

## CONCLUSION

This study aimed to formulate solid microcrystalline dispersion as a buccal film consisting of PVA and PHEA and to evaluate the effect of different PVA concentrations of film properties. When comparing two concentrations of PVA used, DSC and ATR-FTIR results showed similar trends, and no interaction between drug and polymers was observed. However, SEM images showed a smaller particle size for F5 film, and drug release profiles showed a faster dissolution rate during the first 15 min. Result confirmed that formulation of solid microcrystalline dispersions was achieved. Mainly, those were able to increase the drug dissolution rate. F5 and F6 films showed the highest drug release at 15 min, respectively 74% and 72% for F6 film. Therefore, PVA with a concentration of 5% w/v would be ideal when formulating a pharmaceutical film to achieve a fast-release profile. This study showed the suitability of the solvent casting method to produce fast dissolving film and confirmed the relevance of using different concentrations of PVA to improve dissolution profile. Moreover, addiction of PHEA resulted in being of advantage in increasing dissolution profile and regulating drug particle size.
